# Clinical features and survival outcomes of ocular melanoma in a multi-ethnic Asian cohort

**DOI:** 10.1038/s41598-020-73534-x

**Published:** 2020-10-01

**Authors:** Laura Ling Ying Tan, Jiancheng Hong, Wei Lin Goh, Esther Wei Yin Chang, Valerie Shiwen Yang, Eileen Poon, Nagavalli Somasundaram, Mohamad Farid, Anita Sook Yee Chan, Jason Yongsheng Chan

**Affiliations:** 1 Yong Loo Lin School of Medicine, National University of Singapore, Singapore, Singapore; 2 Division of Medical Oncology, National Cancer Centre Singapore, 11 Hospital Drive, Singapore, 169610 Singapore; 3 Institute of Molecular and Cell Biology, Singapore, Singapore; 4 Translational Ophthalmic Pathology Platform, Singapore Eye Research Institute, 20 College Road Discovery Tower, Level 6, The Academia, Singapore, 169856 Singapore; 5 Ocular Inflammation and Immunology Department, Singapore National Eye Centre, Singapore, Singapore; 6 Ophthalmology and Visual Sciences Academic Clinical Program, Duke-NUS Medical School, Singapore, Singapore; 7 Duke-NUS Medical School, Oncology Academic Clinical Program, Singapore, Singapore; 8 Cancer Science Institute of Singapore, National University of Singapore, Singapore, Singapore

**Keywords:** Cancer, Eye cancer

## Abstract

Ocular melanomas are uncommon cancers in Southeast Asia unlike in the West. We conducted a retrospective review of patients (n = 44) with histologically-proven ocular melanoma within a multi-ethnic Asian cohort from Singapore. Clinicopathological features and relapse patterns were examined, and survival outcomes of interest included recurrence-free survival (RFS) and overall survival (OS). Survival analysis was performed using the Kaplan–Meier method and multivariable Cox proportional regression. The study cohort included 18 male and 26 female patients, with a median age of 52 years (range 8–78). Median follow-up was 154 months. For uveal melanomas (n = 29), the 5-year RFS and OS was 56.8% and 76.6%, respectively; whilst for conjunctival melanomas (n = 15), the 5-year RFS and OS was 30.1% and 68.8%, respectively. Fifteen patients (38.5%) eventually developed metastasis, following which the median survival was only 17 months. Multivariate analysis demonstrated that higher T stage was a significant independent predictor for both OS (HR 8.69, 95% CI 1.03 to 73.09, *p* = 0.047) and RFS (HR 11.62, 95% CI 2.45 to 55.00, *p* = 0.002). Smoking history was independently predictive of better RFS (HR 0.08, 95% CI 0.01 to 0.78, *p* = 0.030). In conclusion, our study demonstrates the poor ocular melanoma outcomes in Southeast Asians, highlighting the necessity for urgent research in this area of unmet clinical need.

## Introduction

Ocular melanomas are a group of uncommon cancers^1^, and account for approximately 5% of melanoma cases^2^. More than three-quarters of ocular melanomas involve the uvea, with the remainder of cases arising from the conjunctiva^3^. Uveal melanomas (UM) can arise from any part of the uveal tract, including the choroid, iris and ciliary body. Importantly, while the incidence of UM has remained stable^4^, the incidence of conjunctival melanoma (CM) has risen over the years in the US as well as several countries in Europe^5^, mirroring the increasing incidence of cutaneous melanoma.

Differences in UM and CM are also evident at the molecular level. UMs are characterised by a low mutation burden^1^, with upregulation of the MAPK signalling pathway by an activating mutation in either of the G-proteins *GNAQ* or *GNA11* in up to 80% of cases^1^. Other associated genetic mutations in UM include those involving BRCA1-associated protein (*BAP1*) and rarely, *KIT*^1^. On the other hand, the molecular pathogenesis of CM is more similar to cutaneous and mucosal melanoma and include mutations involving *BRAF*^6^.

Despite these dissimilarities, the management of both types of ocular melanomas is alike and is largely influenced by disease factors including tumour diameter and thickness, degree of extraocular extension, and the presence of metastases. In the absence of metastasis, options for local therapy for UM include radiation therapy or enucleation. For patients with CM, initial management is focused upon wide local surgical excision with adjuvant therapy, such as brachytherapy or localised chemotherapy^4^. In terms of patient outcomes, the 5-year survival rates of localised UM and CM are comparable and approximates 70–80%^7^. Notably, the prognosis for UM depends on molecular characteristics such as monosomy of chromosome 3 and *BAP1* alterations, whereas conventional tumor staging remains the most prognostic for CM^8^.

Epidemiological studies and clinical trials in ocular melanomas have largely been centred on Caucasians^2^ given the significantly higher incidence of ocular melanomas among Caucasians as compared to Asians^4^. In the Collaborative Ocular Melanoma Study (COMS)^9^, 97% of the study population were Caucasian while less than 1% were Asian. The tumour biology and clinical outcomes of ocular melanoma have been proposed to differ across ethnic groups. A retrospective analysis of 8100 UM patients showed that < 1% were Asians (n = 44) and that Asians had a significantly younger age of presentation and higher rate of melanoma-related metastasis at 10 years compared to Caucasians^10^. Asian-specific studies on ocular melanoma at present remain scarce. Asian reports on UM include those from China^11,12^ and South Korea^13,14^, while those on CM include those from China^15,16^ and India^17^. Compared to Western cohorts, Asian patients presented at an earlier age for both UM and CM^11,17^. The prognosis of Asian patients however, vary across studies and remains to be better defined^11,14,15^.

Therefore, we conducted a retrospective review of our patients diagnosed with ocular melanoma across two major tertiary institutions in Singapore, and examined their disease characteristics, prognostic factors and clinical outcomes.

## Materials and methods

### Study cohort

Retrospective review of clinical data of 44 patients with histologically-proven ocular melanoma seen at the National Cancer Centre Singapore (NCCS) and Singapore General Hospital (SGH) from 2004 to 2019 was performed. Clinical and demographic information available included age, sex, ethnicity, presence of symptoms at diagnosis and ECOG performance scores. All histological parameters were reviewed by expert ophthalmic pathologists. Surgical resection margins were determined as R0 (microscopically negative margins), R1 (microscopically positive margins) or R2 (gross residual disease). Where available, data on genetic mutations, systemic chemotherapy, radiotherapy and immunotherapy were included. This work was done under approval from the SingHealth Centralised Institution Review Board. Written informed consent for the use of biospecimens and data was obtained from all participants and/or their legal guardians in accordance with the Declaration of Helsinki. All methods were performed in accordance with the relevant guidelines and regulations. The datasets generated during and/or analysed during the current study are available from the corresponding author on reasonable request. Patient and disease characteristics are summarised in Table 1.

**Table 1:** Clinicopathological characteristics of ocular melanoma patients in the whole study cohort.

Characteristic	Number of cases	%
Total	44	100
*Sex*		
Male	18	40.9
Female	26	59.1
*Age at diagnosis (years)*		
> 40	28	63.6
≤ 40	16	36.4
*Ethnicity*		
Chinese	31	70.5
Non-Chinese	13	29.5
*Performance status (ECOG score)*		
0	33	75
1–3	11	25
*History of smoking*		
Yes	7	15.9
No	37	84.1
*Symptoms at presentation*		
Present	21	47.7
Absent	23	52.3
*Tumour site*		
Uveal	29	65.9
Conjunctival	15	34.1
*Pigmentation*		
High	14	31.8
Low	30	68.2
*Histological type*		
Epithelioid	24	54.5
Non-epithelioid	20	45.5
*Treatment intent*		
Curative^†^	39	88.6
Palliative	3	6.8
Unknown	2	4.5
*AJCC staging (uvea)*		
T1N0M0	2	6.9
T2N0M0	5	17.2
T3N0M0	8	27.6
T4N0M0	6	20.7
T4N1M1	2	6.9
TxN0M0	6	20.7
*AJCC staging (conjunctiva)*		
T1N0M0	7	46.7
T2N0M0	5	33.3
T3N0M0	1	6.7
T4N0M0	1	6.7
T3N1M0	1	6.7

^†^35 patients underwent curative surgery, while 4 patients received radiotherapy alone.

### Statistical analysis

Survival analyses were conducted as described previously^18,19^. Overall survival (OS) was computed from the date of diagnosis to the date of death or last follow-up (for surviving patients). Recurrence-free survival (RFS) was determined by the interval between the date of diagnosis and date of relapse or date of death from any cause, whichever was earliest. Patients who were alive and free of relapse, or lost to follow-up were censored at their date of last follow-up.

Comparisons of the frequencies of categorical variables were performed using Fisher’s Exact test. Kaplan–Meier survival analyses and log rank tests were conducted to identify statistically significant univariable predictors of OS and RFS. This was represented by hazard ratios (HR) and reported with 95% confidence intervals (95% CI). Multivariate Cox regression model via a backwards procedure was employed to determine independence of significant factors identified on univariate analysis. All statistical analyses were conducted assuming a two-sided test with significance level of 0.05 unless otherwise stated, and performed using MedCalc for Windows, version 19.0.7 (MedCalc Software, Ostend, Belgium).

## Results

### Patient demographics and clinical characteristics

Table 1 shows the clinical characteristics of all 44 patients. The mean age was 50 years (median 52; range 8 to 78 years) with 28 patients (63.6%) over 40 years old. There was a female predominance (59.1%). Thirty-one patients (70.5%) were of Chinese ethnicity, with the remaining two patients being of Malay origin, one being of Indian descent and ten comprising a host of other ethnicities. Thirty-three patients (75%) had excellent performance status (ECOG score of 0). In terms of tumour site, the tumour arose from the uvea for twenty-nine patients (65.9%) (choroidal, n = 27; ciliary body, n = 1; iris, n = 1) and from the conjunctiva for the remaining fifteen patients (34.1%) (bulbar, n = 10; palpebral, n = 5). Seven patients (15.9%) had a positive smoking history and twenty-one patients (47.7%) presented with symptoms at diagnosis. The most commonly reported symptoms were blurring of vision (n = 8), pain (n = 5) and decreased visual acuity (n = 4). Redness, pigmentation, floaters, visual field loss, exudation were each reported by 2 patients. Photopsia was reported by 1 patient.

Thirty-nine patients (88.6%) received curative therapy, with 35 patients undergoing surgery as primary treatment, and 4 patients receiving radiotherapy alone. Of the 4 patients who underwent radiotherapy, 2 received brachytherapy while the type of radiotherapy for the remaining 2 patients was unknown. Of the 35 patients who underwent primary surgery, 2 patients with conjunctival melanoma received adjuvant topical mitomycin C therapy.

### Disease characteristics and histopathological features

**Figure 1:**
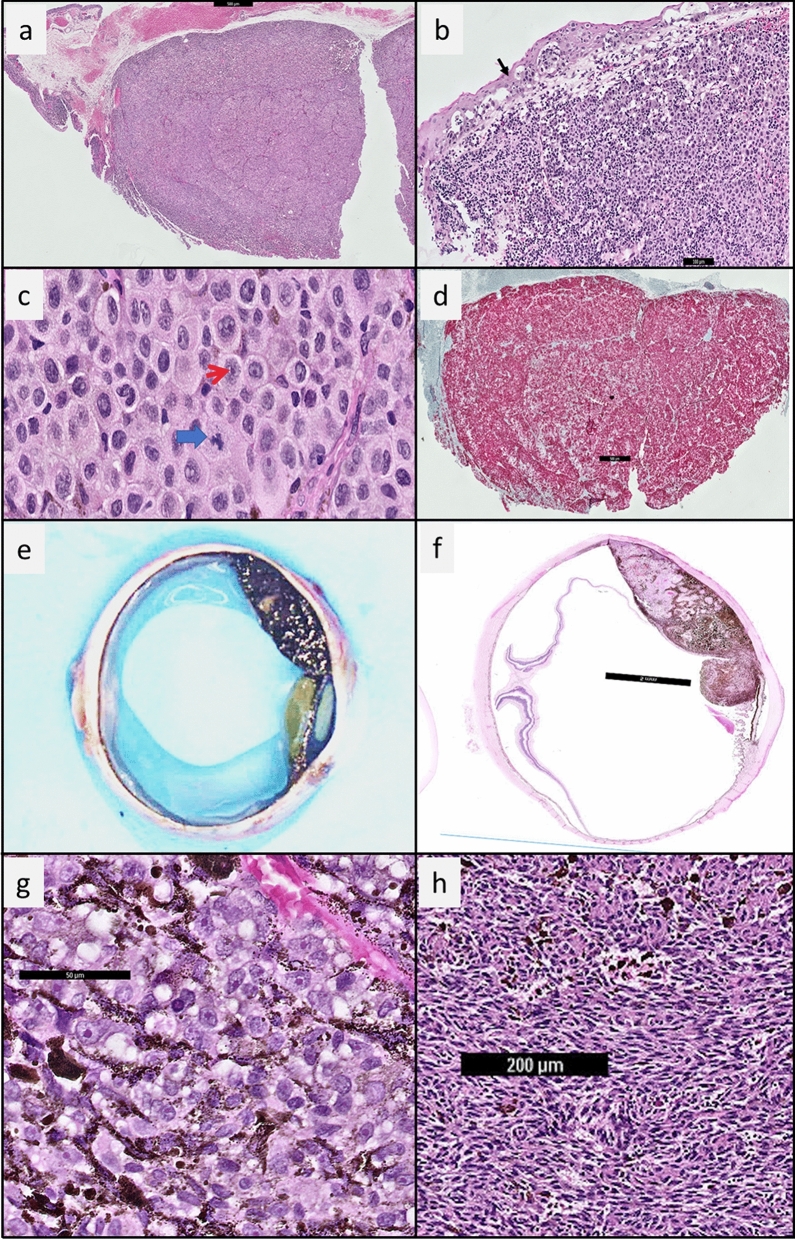
Histology of conjunctiva melanoma in a Chinese woman and histology of uveal melanoma in a Malay man. (**a**) Haematoxylin and Eosin (HE) stain, magnification 2 × . Low power view of an amelanotic conjunctival melanoma. Mutational analysis of this specimen showed the presence of a *BRAF* mutation. (**b**) HE stain, magnification 10 × . Black arrow highlights the pagetoid spread of melanoma cells within the conjunctiva (**c**) HE stain, magnification 40 × . Red arrow highlights epithelioid cells with prominent nucleoli. Blue arrow highlights an atypical mitosis. (**d**) HMB45 immunohistochemistry (IHC) magnification 2 × . The red chromagen for HMB45, a melanocytic marker shows that the tumour cells are melanocytic in lineage. (**e**) Gross image of a pigmented uveal melanoma. This specimen was negative for *BRAF* mutation. (**f**) HE stain, magnification 1 × showing the uveal melanoma extending from the equator of the globe to the ciliary body. (**g**) HE stain, magnification 40 × . Cellular morphology: epithelioid cell melanoma is associated with a poorer prognosis. (h) HE stain, magnification 40 × . Cellular morphology: spindle cell melanoma.

In terms of histopathological features (Table 1), the majority of UM and CM (62.1% and 80%, respectively) were amelanotic (minimal or no pigmentation). Poor prognostic morphology, including epithelioid cells, were more commonly seen in CM (66.7%) than in UM (48%) (Fig. 1). In UM, approximately 51.7% had non-epithelioid spindle cell morphology.

Comparisons between the UM and CM cohorts (Table 2) for all patients (n = 44) revealed a significant difference in presence of symptoms at diagnosis. Eighteen patients (62.1%) with UM were symptomatic, compared with only 3 patients (20%) with CM (*p* = 0.01). Based on the AJCC 7th edition staging for both UM and CM, the extent of primary tumour (T) is categorised based on tumour basal dimension and thickness into four stages (T1 to T4). UM tended to present with more advanced T stages compared to CM (*p* = 0.014). UM presented with larger tumours with 16 of 23 patients presenting with T3 and T4 tumours in comparison to CM where only 3 of 15 patients had T3 and T4 tumours. A subset analysis of comparison between UM and CM patients for non-metastatic ocular melanoma (n = 39) can be found in Table 3.

**Table 2:** Comparison of uveal and conjunctival melanoma in the whole study cohort.

Characteristic	Number of cases (%)		*p*
Total (n = 44)	Uveal	Conjunctival	
*Sex*			
Male	14 (48.3)	4 (26.7)	0.208
Female	15 (51.7)	11(73.3)	
*Age at diagnosis (years)*			
> 40	17 (58.6)	11 (73.3)	0.510
≤ 40	12 (41.4)	4 (26.7)	
*Ethnicity*			
Chinese	22 (75.9)	9 (60)	0.313
Non-Chinese	7 (24.1)	6 (40)	
*Performance status (ECOG score)*			
0	21 (72.4)	12 (80)	0.722
1–3	8 (27.6)	3 (20)	
*History of smoking*			
Yes	6 (20.7)	1 (6.7)	0.393
No	23 (79.3)	14 (93.3)	
*Symptoms at presentation*			
Present	18 (62.1)	3 (20)	0.011
Absent	11 (37.9)	12 (80)	
*Pigmentation*			
High	11 (37.9)	3 (20)	0.314
Low	18 (62.1)	12 (80)	
*Histological type*			
Epithelioid	14 (48.3)	10 (66.7)	0.342
Non-epithelioid	15 (51.7)	5 (33.3)	
*Molecular status of BRAF*			
Mutant	1 (8.3)	4 (57.1)	0.038
Wildtype	11 (91.7)	3 (42.9)	
*Tumour depth*			
T1	2 (8.7)	7 (46.7)	0.014
T2	5 (21.7)	5 (33.3)	
T3	8 (34.8)	2 (13.3)	
T4	8 (34.8)	1 (6.7)

**Table 3:** Comparison of uveal and conjunctival melanoma treated with curative intent.

Characteristic (n)	Number of cases (%)		*p*
Total (n = 39)	Uveal	Conjunctival	
*Sex*			
Male	13 (52)	3 (21.4)	0.093
Female	12 (48)	11 (78.6)	
*Age at diagnosis (years)*			
> 40	13 (52)	11 (78.6)	0.171
≤ 40	12 (48)	3 (21.4)	
*Ethnicity*			
Chinese	19 (76)	9 (64.3)	0.478
Non-Chinese	6 (24)	5 (25.7)	
*Performance status (ECOG score)*			
0	21 (84)	11 (78.6)	0.481
1	3 (12)	2 (14.3)	
2	1 (4)	0 (0)	
3	0 (0)	1 (7.1)	
*History of smoking*			
Yes	6 (24)	1 (7.1)	0.386
No	19 (76)	13 (92.9)	
Symptoms at presentation			
Present	16 (64)	3 (21.4)	0.019
Absent	9 (36)	11 (78.6)	
*Pigmentation*			
High	10 (40)	3 (21.4)	0.304
Low	15 (60)	11 (78.6)	
*Histological type*			
Epithelioid	13 (52)	10 (71.4)	0.317
Non-epithelioid	12 (48)	4 (28.6)	
*Molecular status of BRAF*			
Mutant	0 (0)	4 (57.1)	0.019
Wildtype	9 (100)	3 (42.9)	
*Tumour depth*			
T1	2 (10.0)	6 (42.9)	0.057
T2	5 (20.0)	5 (35.7)	
T3	7 (35.0)	2 (14.3)	
T4	6 (30.0)	1 (7.1)	

### BRAF and CKIT mutation status

Results of molecular tests were only available for selected patients as these were not routinely performed unless they had developed metastatic disease requiring systemic therapy (Table 4). *BRAF* mutation status was known for nineteen patients (n = 12 for uveal and n = 7 for conjunctival), of which five were positive for *BRAF* mutation. Most cases (14 of 19) had relapsed with distant metastasis There was a significant difference in the detection of *BRAF* mutant status between UM (1 of 12, 8.3%) and CM patients (4 of 7, 57.1%) (*p* = 0.038). Four patients with CM had *BRAF* V600E (T1799A) mutation confirmed via Sanger sequencing. The remaining patient with UM of choroidal origin harboured a S607F mutation.

**Table 4:** Molecular status of ocular melanomas.

Gene (cases tested)	Number of cases	
	Uveal (%)	Conjunctival (%)
*BRAF* mutation (19)		
V600E	0 (0)	4 (57.1)
S607F^†^	1 (8.3)	0 (0)
Not detected	11 (91.7)	3 (42.9)
*CKIT* mutation^‡^ (13)		
Exon 11	1 (10)	0 (0)
Not detected	9 (90)	3 (100)

^†^Lies within protein kinase domain of the Braf protein but actual effect on protein function remains unknown^36^.

^‡^Two point mutations at codon 558 (AAG to AAT) and codon 559 (GTT to ATT). Exons 9 and 11 were exclusively tested in 4 cases, an extended panel of exons 9, 11, 13 and 17 was tested in 7 cases. 1 patient was tested via a targeted gene panel while another was unknown.

*CKIT* mutation status was evaluated in thirteen patients (n = 10 for uveal and n = 3 for conjunctival). Exons 9 and 11 were exclusively tested in 4 cases, an extended panel of exons 9, 11, 13 and 17 was tested in 7 cases. 1 patient was tested via a targeted gene panel while another was unknown. Only one patient with UM tested positive for *CKIT* point mutations in codons 558 (AAG to AAT) and 559 (GTT to ATT).

### Survival analyses

**Figure 2:**
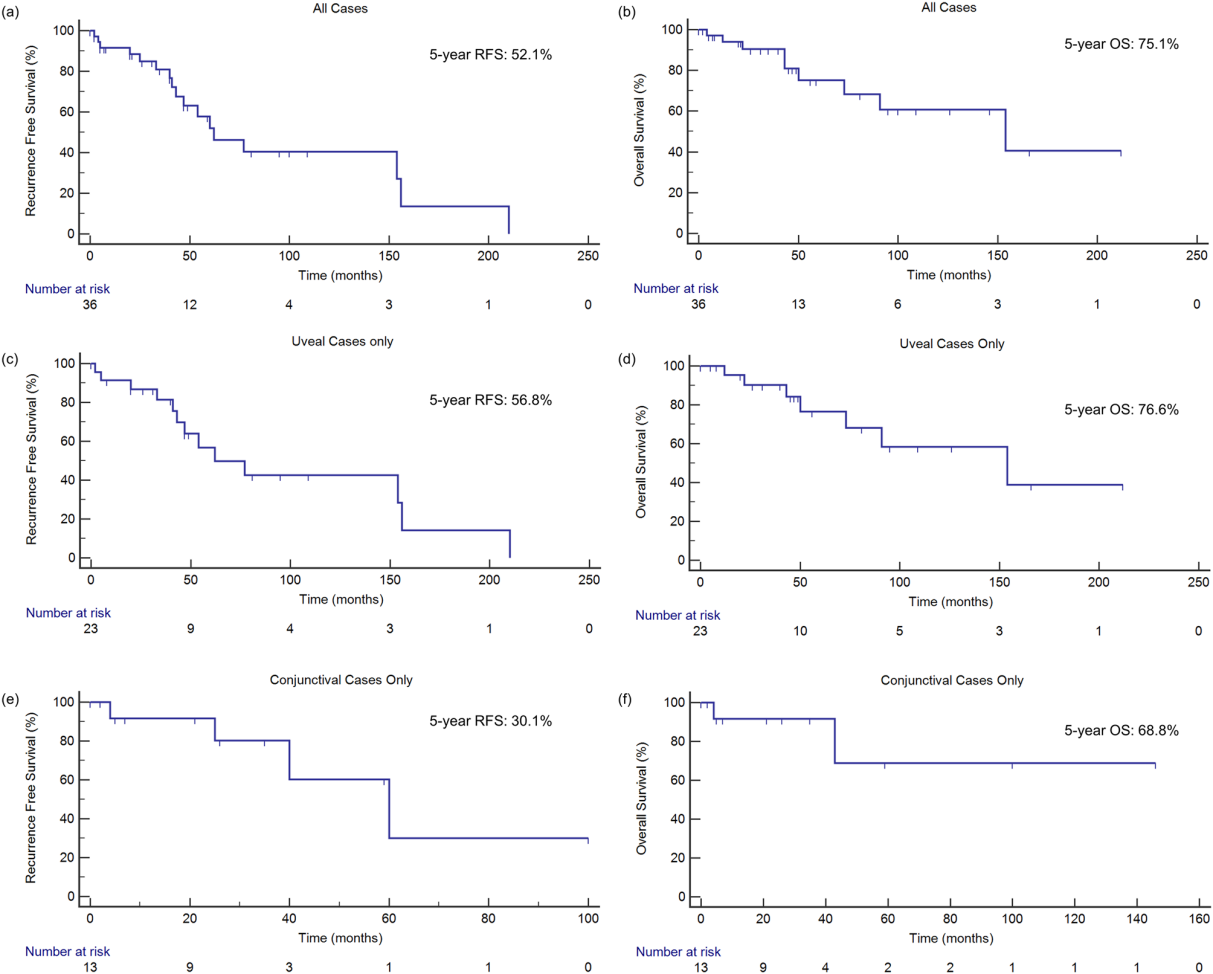
Overall and recurrence-free survival for non-metastatic ocular melanoma. For all non-metastatic ocular melanoma cases (n = 39), (**a**) 5-year RFS was 52.1%, (**b**) 5-year OS was 75.1%. For uveal cases only (n = 25), (**c**) 5-year RFS was 56.8% and (**d**) 5-year OS was 76.6%. For conjunctival cases only (n = 14), (**e**) 5-year RFS was 30.1% and (**f**) 5-year OS was 68.8%.

Survival analyses were performed for patients with non-metastatic ocular melanoma who underwent curative treatment (n = 39). The median follow-up time was 154 months. At the time of analysis, 23.1% of patients (n = 9) had died. The cause of death was related to metastatic melanoma in seven patients and other causes in the remaining patients (n = 1 from ischemic heart disease and n = 1 from metastatic colorectal cancer). The 5-year RFS and OS for all non-metastatic cases was 52.1% and 75.1% respectively. The 5-year RFS and OS for UM (n = 25) was 56.8% and 76.6% respectively while the 5-year RFS and OS for CM (n = 14) was 30.1% and 68.8% respectively (Fig. 2).

Fifteen patients (38.5%) eventually relapsed with metastatic disease. A single site of metastasis was identified in 5 patients, while multiple sites of metastases were identified in the remaining 10 patients. The most common site of metastasis was the liver (n = 11). Metastasis to the subcutaneous tissue, bone, and lymph node were each identified in 3 patients. Metastasis to the peritoneum, spleen and adrenal gland were each identified in 2 patients. Metastasis to the lung, pancreas, vertebrae, brain and kidney were each identified in a single patient.

**Figure 3:**
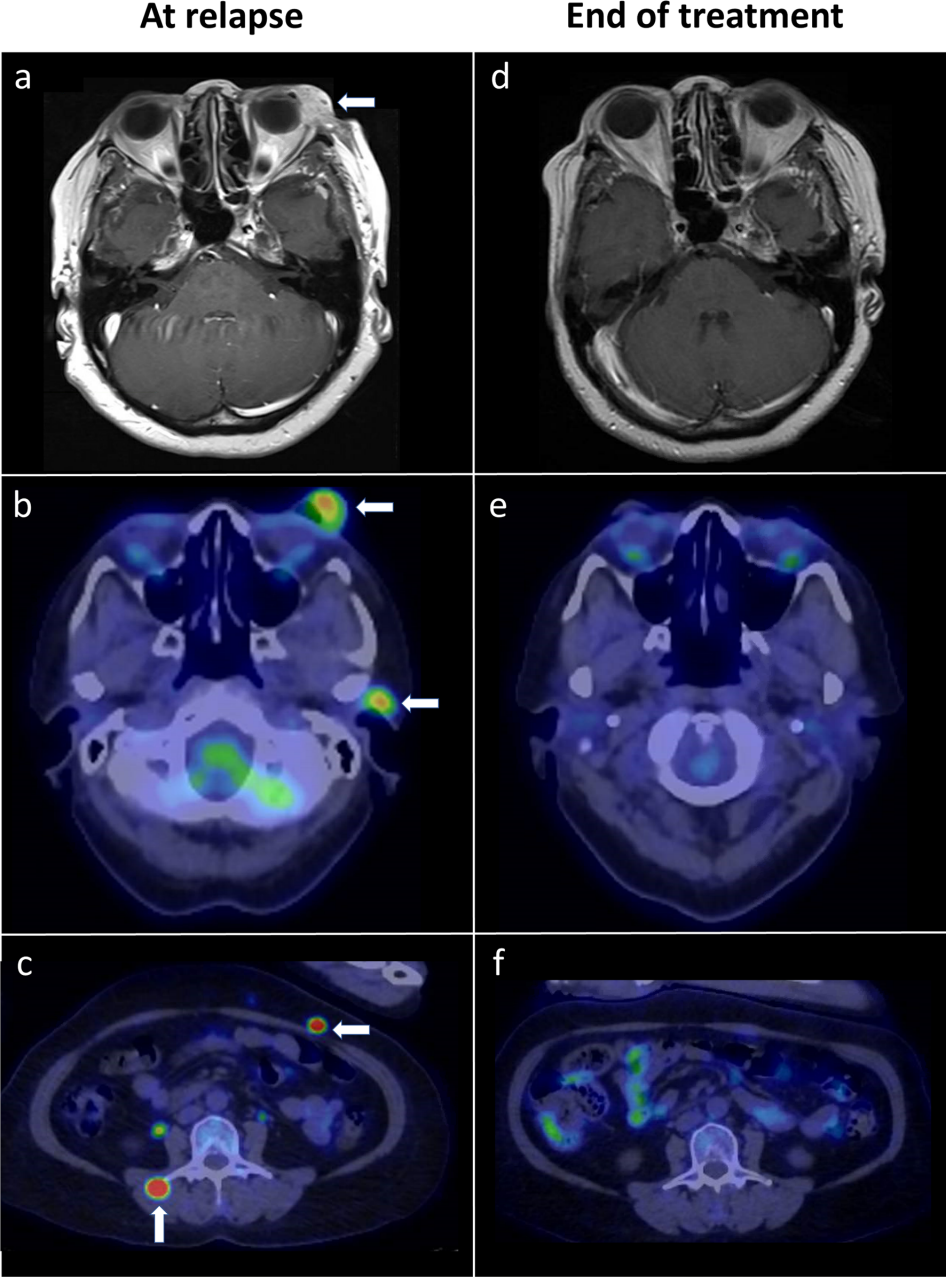
Complete response to Pembrolizumab in recurrent metastatic conjunctival melanoma. (**a**) MRI brain showing a large enhancing mass arising from the left conjunctiva enveloping the left lacrimal gland, at the point of relapse before treatment. (**b**) Corresponding PET-CT images demonstrating hypermetabolic tumours of the left conjunctiva, left parotid lymph node, (**c**) abdominal wall as well as erector spinae muscles. (**d**–**f**) Complete radiological and metabolic response was achieved after 2 years of Pembrolizumab.

Of the fifteen patients who relapsed, the median OS from the time of relapse was 17 months with a 1-year OS of 54.4%. Five patients were lost to follow-up shortly after diagnosis of relapse. Six received supportive care alone and six received systemic therapies with pembrolizumab (n = 2), dacarbazine (n = 1), temozolomide (n = 1), imatinib (n = 1), and nivolumab followed by paclitaxel and carboplatin (n = 1). All, except a single patient who received pembrolizumab, had UM. Treatment data was not available for three patients. Tumour response to systemic therapy was assessed using the RECIST criteria. Of the two patients treated with pembrolizumab, one developed progressive disease (UM) and the other (CM) achieved complete remission. The CM patient received 100 mg of Pembrolizumab every 3 weeks and achieved complete radiological and metabolic remission after 2 years of therapy, and remained alive 86 months from the diagnosis of metastasis (Fig. 3). The patient treated with nivolumab experienced disease progression but subsequently achieved stable disease on paclitaxel and carboplatin. The patient treated with dacarbazine developed disease progression and died from metastatic malignant melanoma 10 months later. The patient with *CKIT* exon 11 mutant UM received imatinib but died from disease one month after. The response to temozolomide was unknown due to loss to follow-up. Interestingly, one patient with UM had indolent lung-only metastasis and remained alive at 48 months from relapse while on expectant care alone.

**Figure 4:**
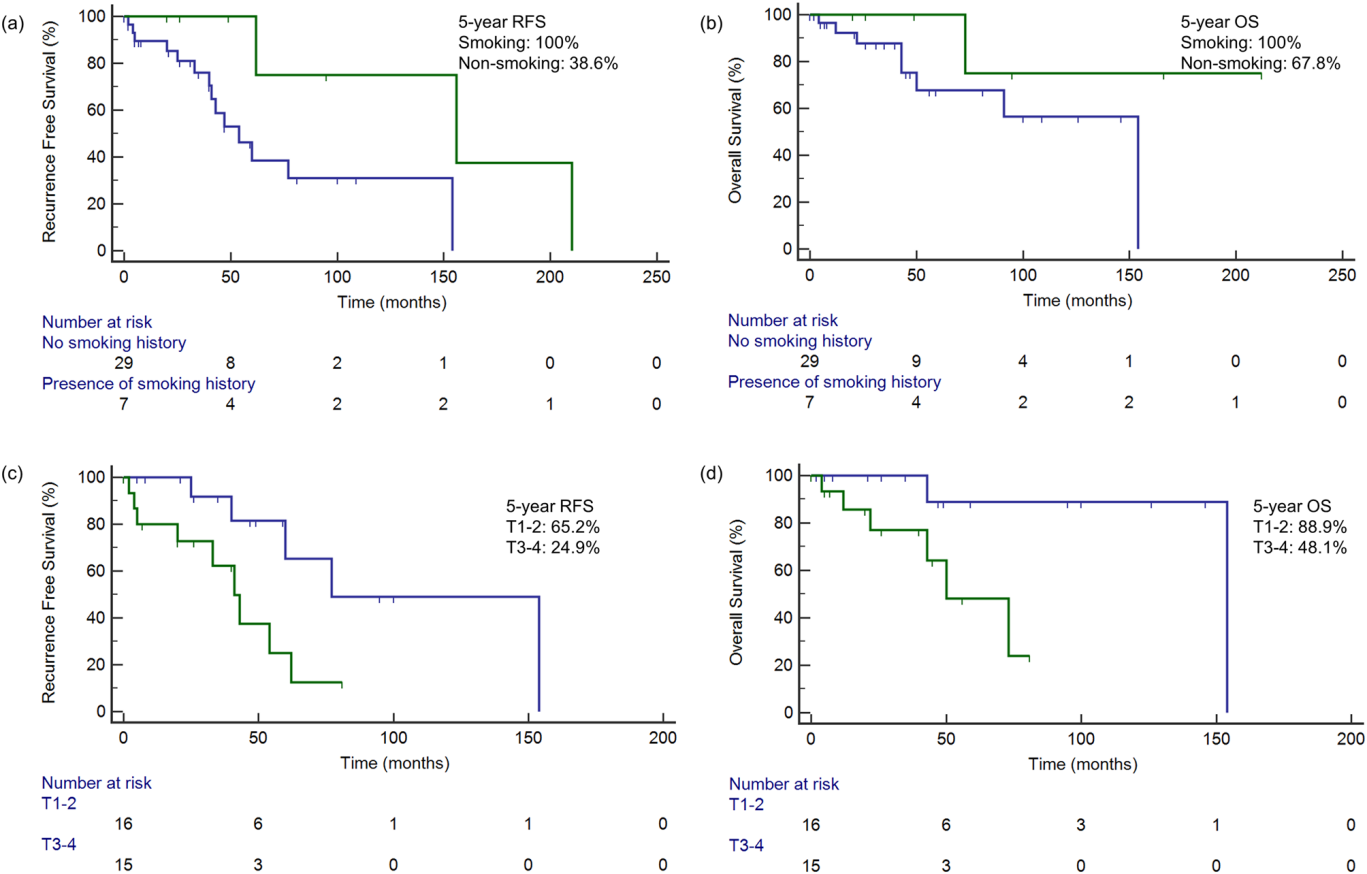
Overall and recurrence-free survival stratified by positive smoking history and presence of symptoms at diagnosis. (**a**) The 5-year RFS in patients with positive and no smoking history was 100% and 38.6% respectively. (**b**) 5-year OS for patients with a positive and no smoking history were 100% and 67.8%. (**c**) The 5-year RFS in patients with T1–2 disease compared to T3–4 was 65.2% and 24.9% respectively. (**d**) 5-year OS in patients with T1–2 disease compared to T3–4 was 88.9% and 48.1% respectively.

Univariate analysis (Table 5) showed that higher T stage (T3 and T4 disease) was significantly associated with worse RFS (HR 3.81, 95% CI 1.23 to 11.83, *p* = 0.021) and OS (HR 6.56, 95% CI 1.42 to 30.35, *p* = 0.016). Increased pigmentation on histological examination was associated with worse RFS (HR 3.27, 95% CI 1.12 to 9.59, *p* = 0.031) but the association with OS was not statistically significant (HR 1.60, 95% CI 0.40 to 6.43, *p* = 0.510). Interestingly, a positive smoking history was significantly correlated with better RFS (HR 0.29, 95% CI 0.09 to 0.90, *p* = 0.032). Analyzing the effect of smoking on UM separately, we found that the protective effect on RFS was still observed (HR 0.26, 95% CI 0.08 to 0.90, *p* = 0.034). Multivariate analysis (Table 6) demonstrated that higher T stage was a significant independent predictor for both OS (HR 8.69, 95% CI 1.03 to 73.09, *p* = 0.047) and RFS (HR 11.62, 95% CI 2.45 to 55.00, *p* = 0.002). Likewise, a positive smoking history remained a significant independent predictor of RFS (HR 0.08, 95% CI 0.01 to 0.78, *p* = 0.030), as well as age > 40 years (HR 8.58, 95% CI 1.69 to 43.48, *p* = 0.009). The 5-year RFS and OS for patients with a positive smoking history were both at 100% compared to 38.6% and 67.8% respectively in never smokers. The 5-year RFS and OS in patients with T1–2 disease were 65.2% and 88.9% respectively, compared to 24.9% and 48.1% in patients with T3–4 disease (Fig. 4).

**Table 5:** Univariate survival analysis for ocular melanoma treated with curative intent.

Characteristic		Recurrence-free survival		Overall survival	
		HR (95% CI)	*p*	HR (95% CI)	*p*
Sex	Female	Referent	0.163	Referent	0.181
	Male	0.49 (0.18, 1.33)		0.40 (0.10, 1.53)	
Age at diagnosis (years)	≤ 40	Referent	0.276	Referent	0.117
	> 40	1.76 (0.64, 4.84)		2.99 (0.76,11.79)	
ECOG performance status	0	Referent	0.945	Referent	0.696
	1–3	0.96 (0.26 to 3.43)		1.42 (0.24, 8.25)	
History of smoking	No	Referent	0.032	Referent	0.167
	Yes	0.29 (0.09, 0.90)		0.35 (0.08, 1.55)	
Symptoms at presentation	Absent	Referent	0.087	Referent	0.143
	Present	2.52 (0.87, 7.27)		2.88 (0.70, 11.82)	
Tumour site	Conjunctiva	Referent	0.686	Referent	0.986
	Uvea	0.77 (0.22, 2.69)		0.99 (0.19, 5.02)	
Pigmentation	Low	Referent	0.031	Referent	0.510
	High	3.27 (1.12, 9.59)		1.60 (0.40, 6.43)	
Histological type	Non-epithelioid	Referent	0.245	Referent	0.549
	Epithelioid	1.80 (0.67, 4.84)		1.51 (0.40, 5.73)	
AJCC staging	T1–2	Referent	0.021	Referent	0.016
	T3–4	3.81 (1.23, 11.83)		6.56 (1.42, 30.35)

**Table 6:** Multivariate analysis^†^ for ocular melanoma treated with curative intent.

Characteristic		Recurrence-free survival		Overall survival	
		HR (95% CI)	*p*	HR (95% CI)	*p*
Age at diagnosis (years)	≤ 40	Referent	0.009	–	–
	> 40	8.58 (1.69, 43.48)			
History of smoking	No	Referent	0.030	–	–
	Yes	0.08 (0.01, 0.78)			
AJCC staging	T1–2	Referent	0.002	Referent	0.047
	T3–4	11.62 (2.45, 55.00)		8.69 (1.03, 73.09)	

^†^Covariates included those *p* < 0.3 on univariate analysis using a backward model.

## Discussion

This study analyses the clinical outcomes of ocular melanoma in Southeast Asia. The mean age at presentation in our entire cohort was 50 years, with a mean age of 48 and 52 for UM and CM respectively. This is similar to previous studies on UM in Chinese and Koreans which reported an earlier age of presentation for UM (mean age 48 to 53 years)^11,13^ compared to Caucasians (mean age 60 years)^9^. For CM, Chinese and Indian patients also have a younger age at presentation (mean age 43 to 54 years)^15,17^ compared to Caucasians (mean age 55 to 65 years)^6^. Survival analysis in our study was focused on patients with non-metastatic disease as tumour behaviour and prognostic factors of primary non-metastatic and metastatic disease differ greatly^20^. The 5-year RFS and OS for UM (n = 25) was 56.8% and 76.6% respectively in our multiracial population. Previous reports suggested the survival rate in Asians with UM to be higher than their Caucasian counterparts, which has been postulated to be due to the younger age of presentation in Asians^11^. In Chinese (n = 171) and Korean (n = 33) patients, the 5-year metastasis-free survival rate approximates 80%^11,14^ and are higher survival rates than European (n = 5,788)^21^ and US cohorts (n = 1,003)^22^ which reported 5-year survival rates bordering at or less than 70%^11^. However, these lower survival rates were based on older data and more recent studies in the US^23^ and Scotland (n = 218)^24^ report slightly higher 5-year cancer-specific survival ranging from 70 to 80%. On the contrary for CM, poorer survival outcomes have been reported in Asians compared to Caucasians, due in part to the advanced stages at presentation and poor melanoma awareness in Asia^15,17^. In our cohort, the 5-year RFS and OS for CM (n = 14) are 30.1% and 68.8%, respectively. Studies in China (n = 57)^15^ and India (n = 42)^17^ report 5-year tumour-related death rates at 30.5% and 11% respectively. CM outcomes in Caucasian populations vary with 5-year cancer-specific survival ranging from 93% in the US^25^ to 86.3% in the Netherlands (n = 194)^26^.

The development of metastatic disease in patients with ocular melanoma, specifically UM, portends a bleak clinical scenario with few therapeutic options and dismal prognosis. In our study, over a third of the patients with localised disease eventually relapsed with metastatic disease, with a median OS of 17 months and 1-year OS of 54.4%. Half of these patients were subsequently placed on systemic therapy. Tumour response to these therapies were poor with most patients in our cohort experiencing progressive disease except for one patient achieving disease stability from paclitaxel and carboplatin. Interestingly though, one patient had indolent lung-only metastasis and remained alive 4 years following relapse while only receiving supportive care. Clinical outcomes after metastasis for UM has been reported to be similarly poor in other Asian cohorts, ranging from a median OS of 5 months to 15.7 months^13,14^. The identification of patients at risk of relapse for close follow-up and early intervention may be necessary to overcome this unmet clinical need.

Interestingly, we observed that the presence of symptoms at diagnosis was significantly associated with UM as compared to CM. The most common symptom at presentation was blurring of vision, which is consistent with previous studies in Caucasian cohorts^27,28^. Previous studies have similarly indicated that the majority of UM patients are symptomatic at the time of diagnosis^4^ while CM is often asymptomatic^6^. UM is more likely to affect vision especially if within the visual axis as opposed to tumours arising from the bulbar conjunctiva, the most common location of CM. Bulbar CM may be present as a cosmetic non-issue that in our pigmented population may be tolerable. Additionally, our study also showed they tended to be less pigmented and hence even less of a cosmetic issue. Bulbar CM, especially those not reaching the limbus, may also remain hidden beneath the eyelid or fornix and remain asymptomatic till the mass is large^25^.

Additionally, our results suggest that a positive smoking history is independently associated with better RFS. Though smoking is a known risk factor for many cancers, epidemiological studies and meta-analyses have reported a decreased risk of cutaneous malignant melanoma^29,30^ and intraocular malignant melanoma^31^ among smokers. Smoking is known to protect the skin from ultraviolet (UV) radiation-induced inflammation^31^, which occurs due to the chronic effect of nicotine accumulation in melanocytes^32^. Transdermal application of nicotine is known to suppress the cutaneous inflammatory response to ultraviolet B radiation^33^. Studies exploring specifically UM indicate however, that smoking does not alter the risk of early metastases^34^ nor does it confer a significant increase in long-term risk of the development of secondary cancer^35^. The mechanisms by which smoking affects the risks of incident melanomas may also be different from those modulating disease relapse. Taken together, the potential protective association of smoking and survival outcome of ocular melanoma remains to be verified in future studies.

Our study does have certain limitations as a retrospective study with inherent selection biases. Given the small sample size, confidence intervals of effect sizes observed were wide and the possibility of chance findings cannot be excluded. In addition, information on subsequent relapse and treatment may not be available for patients lost to follow-up. The lack of genetic information in UM was also another limitation as this is not widely available in this region until recently. Nonetheless, the strength of our study is the focus on the clinical characteristics, prognostic factors and survival outcomes of ocular melanoma in Southeast Asians which is rarely explored in large ocular melanoma studies.

In conclusion, our study demonstrates the poor outcomes of ocular melanoma in Southeast Asians, highlighting the need for urgent research in this area of unmet clinical need.
